# Professionals' and non‐professionals' experiences of working with people with Hoarding Disorder: A thematic synthesis

**DOI:** 10.1111/bjc.70022

**Published:** 2025-11-28

**Authors:** Hannah Parker, Louise Waddington, Bethan Shergold, James D. Gregory

**Affiliations:** ^1^ School of Psychology Cardiff University Cardiff UK

**Keywords:** Hoarding Disorder, systematic reviews, psychological interventions, psychological disorders, qualitative, therapeutic alliance/therapeutic factors

## Abstract

**Objectives:**

Individuals with Hoarding Disorder can encounter a range of professionals and non‐professionals during the course of receiving support to manage the accumulation of belongings. This thematic synthesis explored the experiences of professionals and non‐professionals working with people with Hoarding Disorder.

**Methods:**

PsycInfo, Embase and PubMed databases were searched in October 2023. Published and unpublished studies were included. Search terms covered various helping professions including volunteer support, and qualitative designs, in relation to Hoarding Disorder. This identified 12 studies. All text within the study ‘Results’ or ‘Findings’ section was extracted for data synthesis to capture both author interpretations and participant quotations (mixed method studies extracted qualitative data only). Data were coded ‘line by line’, which led to descriptive themes being created followed by analytical themes.

**Results:**

Four themes were identified: (1) Relationships are Complex; (2) Possessions are Just the Tip of the Iceberg; (3) Juggling Dilemmas on a Tightrope; and (4) Working with Hoarding is Like a Puzzle to Solve. Twelve subthemes were also identified. These themes described the various challenges involved in working with Hoarding Disorder, including the role of relationships with services and others, comorbidities, such as trauma and executive dysfunction, the ethical dilemmas which are apparent during the work, and the differing perceptions of working with Hoarding Disorder.

**Conclusions:**

This thematic synthesis identifies common challenges involved in working with Hoarding Disorder and offers recommendations as to how professionals and non‐professionals can approach their work with this client group.

**Practitioner points**

- Working with HD appears to be challenging for workers across several domains.
- There is a need to adapt practice and work creatively with people with HD.
- Support for professionals and non‐professionals, such as regular supervision, is vital to prevent burnout.

## INTRODUCTION

Hoarding Disorder (HD) is a mental health condition characterised by difficulties in discarding possessions leading to the build‐up of clutter, which affects the use of the main living areas of the home (American Psychiatric Association [APA], 2013). Given the elevated rates of comorbid physical and mental health problems (Bates et al., 2021; Frost et al., 2011; Tolin et al., 2011), as well as a history of trauma and executive functioning difficulties (Ayers et al., 2013; Landau et al., 2011), HD is a complex mental health problem. The complexity of HD is further reflected in difficulties with treatment engagement and modest outcomes following psychological intervention (Tolin et al., 2007, 2015). Cognitive behavioural therapy (CBT) is the leading evidence‐based psychological therapy for HD. A recent meta‐analysis of CBT for HD demonstrated large effect sizes, with outcomes maintained in the small number of studies that offered follow‐ups (Rodgers et al., 2021), although with no follow‐ups after 6 months it is unclear whether progress is observed in the longer term. Furthermore, rates of clinically significant change across group and individual CBT are low, varying between 25% and 43% (Tolin et al., 2015), in comparison to the higher rates seen in obsessive compulsive disorder (OCD) (46–76%; Abramowitz et al., 2003). We also know that people with HD display ambivalence prior to and during treatment (Steketee et al., 2010), there can be high drop‐out rates (Mataix‐Cols et al., 2002; Tolin et al., 2007), and that maintaining motivation is an ongoing challenge (Ayers et al., 2015).

As well as psychological therapy, HD often requires involvement from professionals working across an array of other disciplines, such as housing officers, social workers and environmental health, due to the wide‐ranging impact on the individual, their living environment and those around them. Psychosocial interventions may have outcomes comparable to CBT (Twigger et al., 2024) and may be in the form of harm reduction approaches which place emphasis on fire prevention and maintaining tenancy, such as the Hoarding Action Response Team (HART) model (Kysow et al., 2020). However, these are subject to similar difficulties with engagement, with clients missing appointments and withdrawing their consent for intervention.

People with HD may also receive assistance from non‐professionals such as charities and organisations offering volunteer‐led support (Ryninks et al., 2019). Volunteers have been a useful adjunct to group CBT for HD by supporting with decluttering in the home, with participants highly satisfied with the convenience of support offered (Crone et al., 2020), however the number of sessions was restricted to eight, and participants expressed only moderate satisfaction with the personalisation of the programme and the level of accountability they were subject to.

Given the challenges with engagement observed across a range of interventions, it is important that factors which may affect acceptability are considered, such as the type and nature of support offered. In a study exploring the acceptability of interventions (Rodriguez et al., 2016), only 3 out of 11 interventions were deemed acceptable by people with HD (individual CBT, professional organising service, self‐help book) and less than a third of participants had tried any intervention. This may be due to the ego‐syntonic nature of HD, with people describing the comfort and sense of safety which their belongings offer them (Mulligan‐Rabbitt et al., 2023).

People with HD also value the interpersonal aspects of intervention. In a collective case study of older adults receiving CBT for HD, the majority referred to ‘encouragement, kindness, and support’ as being a useful component of therapy, with the relationship deemed to be critical for treatment completion (Ayers et al., 2012). The importance of these common factor skills is also supported by other professional disciplines; for example, social workers emphasise the forging of good relationships with people with HD by being non‐judgemental, patient and empathetic, and recognising that this process can take time (Brown & Pain, 2014). Occupational therapy staff are also aware of the need to build rapport with people with HD, as well as encouraging autonomy (Kretzer et al., 2023). Furthermore, recently published good practice guidelines for hoarding from The British Psychological Society (2024, p. 33) state ‘it is important to remain non‐judgemental while holding on to hope that things can change’.

However, research has identified contrasting approaches across professionals when working with HD, with some prioritising the therapeutic relationship, through to taking a more pragmatic approach, and expressing shock and frustration (Holden et al., 2019). This may be reflective of how professional involvement may be due to necessity rather than at the request of the individual with HD; most requests for help with hoarding difficulties come from family members or mental health professionals compared to just one‐third of enquiries coming from the individual themselves (Bratiotis et al., 2016). Professionals report challenges in working with people with HD such as lack of insight and defensiveness (Frost et al., 2010), as well as feelings of frustration and irritation (Tolin et al., 2012).

These concerns are reflected in the descriptions of help‐seeking in individuals with HD, with themes centred around difficulties in relating to hoarding as a problem, fear of being labelled and judged, and experiencing gaps in service provision, as well as reporting feeling rejected by services and finding it hard to trust others (McGrath et al., 2024). These factors are likely to be disruptive to the development of the therapeutic relationship and act as a significant barrier to people with HD first accessing, and subsequently, engaging in the support they need, due to interconnection being perceived as key by people with HD in reducing their hoarding symptoms and positively affecting wellbeing (Jones et al., 2025). Poor treatment experiences are also likely to compound the internalised stigma which people with HD have described, which may negatively impact upon help‐seeking (Prosser et al., 2024). People with HD have reported that they would like professionals to have a better understanding of hoarding, ‘reduced judgement and stigma’ and better working across professionals (French et al., 2022).

Despite HD requiring input from a range of professionals and non‐professionals, and the literature indicating the importance of effective working relationships in supporting people with HD, to date there has not been a review of what the experience of supporting people with HD is like. Gaining a better insight into these experiences may enhance understanding of how to support people with HD, including issues such as uptake of treatment, engagement and attrition. This could also help those working with people with HD feel more prepared and have a better idea of what to expect when working with this population. This systematic review aims to answer the following question: What are the experiences of professionals and non‐professionals working with people with HD?

## METHOD

The systematic review followed PRISMA (Preferred Reporting Items for Systematic reviews and Meta‐Analyses) reporting guidelines (Page et al., 2021), including pre‐registration on PROSPERO (International Prospective Register of Systematic Reviews; Registration Number: CRD42023391664). The research question was formulated using the PICo (Population, Phenomena of Interest, Context) framework (Stern et al., 2014). The population was defined as professionals and non‐professionals supporting adults with HD. The phenomenon of interest was experiences of providing support to people with HD, with no restrictions on the nature of the support offered and this taking place in any context.

### Search strategy

PsycInfo, Embase and PubMed databases were searched in October 2023. Search terms can be found in Table 1. Reference lists of eligible studies were also screened. Unpublished studies (e.g., doctoral theses) were also included. Email contact was made with leading authors in the field to seek additional studies. There was no restriction on when the studies were published.

**TABLE 1:** Search terms used to identify studies related to experiences of working with hoarding.

Search term definitions	Search terms
Population	(hoard*) AND
Type of Professional/Non‐Professional	(policing OR police* OR officer* OR fire* OR ‘environmental health’ OR therapist* OR consultant* OR doctor* OR GP OR ‘general practitioner*’ OR professional* OR volunteer* OR psychologist* OR ‘social worker*’ OR ‘occupational therapist*’ OR nurse* OR housing OR landlady OR landlord* OR manager* OR multi* OR team*) AND
Type of Qualitative Study	(narrative or template or discourse or ‘Interpretative phenomenology*’ OR ‘grounded theory’ OR ‘thematic’ OR ‘content’ OR ‘phenomenological approach’ OR ‘constructivist epistemological framework’ OR structured OR semi structured OR unstructured OR informal OR in‐depth OR ‘face‐to‐face’ OR guide* OR interview* OR discussion* OR questionnaire* OR ‘focus group’ OR qualitative OR ethnograph* OR ‘field work’ OR fieldwork OR ‘key informant’)

References were uploaded to EndNote 20 and de‐duplicated, then exported to Rayyan (systematic review organisational website tool) for further de‐duplication. The title and abstract screening was then carried out by the lead researcher (H.P.) using the inclusion and exclusion criteria (see Table 2). 10% of the title and abstracts were screened by a second reviewer (B.S.), with this selection split proportionally across those papers being taken forward to full text screening (those labelled as ‘Yes’ or ‘Maybe’) and those being excluded, which led to 100% inter‐rater reliability.

**TABLE 2:** Inclusion/Exclusion criteria for studies related to experiences of working with hoarding.

	Criteria
Inclusion	Professionals and non‐professionals describing experiences of supporting adults with HD (either self‐reported or identified using diagnostic criteria, e.g., Diagnostic and Statistical Manual of Mental Disorders [DSM‐5], APA, 2013, International Classification of Diseases [ICD‐11], World Health Organization, 2019) Qualitative studies Mixed method studies, but only qualitative data to be extracted English language articles only
Exclusion	Studies focusing on animal hoarding only

**FIGURE 1:**
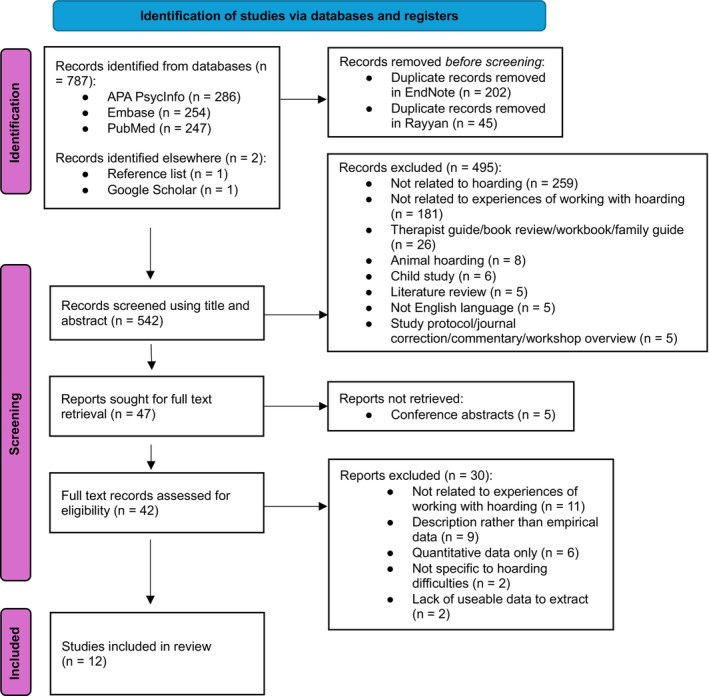
PRISMA Diagram Identifying Study Selection for Thematic Synthesis.

Papers at the full text stage were screened by the lead researcher. When it was uncertain whether a paper met the inclusion/exclusion criteria, this was discussed with another member of the review team (J.G.) until agreement was reached. At the full text stage, a second reviewer (B.S.) read 20% of the papers; again this was split proportionally across included and excluded papers, with 100% inter‐rater reliability. Study selection is outlined using a PRISMA diagram (Page et al., 2021; see Figure 1).

### Data extraction

Data were documented using a modified data extraction form (Munro et al., 2007). Examples of the type of data which were extracted include method of identifying HD, professionals' and/or non‐professionals' roles and key characteristics, and data analysis method.

### Quality assessment

The CASP (Critical Appraisal Skills Programme) Qualitative Studies Checklist (CASP, 2024) was used as a quality appraisal tool. This features 10 questions across three domains regarding the validity, rigour and helpfulness of the results, which are answered ‘Yes’, ‘No’ or ‘Can't tell’. The CASP Checklist does not feature a formula for rating quality; therefore, the National Institute for Health and Care Excellence (NICE, 2012) quality rating tool was applied (see Table 3), which provides a rating based on the volume of checklist criteria which have been met. The lead researcher assessed quality appraisal, and 20% of papers were checked by additional members of the review team to corroborate the ratings, which gave 100% inter‐rater reliability.

**TABLE 3:** NICE (2012) quality rating tool.

Quality rating	Quality rating criteria
++	All or most of the checklist criteria have been fulfilled, where they have not been fulfilled the conclusions are very unlikely to alter.
+	Some of the checklist criteria have been fulfilled, where they have not been fulfilled, or not adequately described, the conclusions are unlikely to alter.
−	Few or no checklist criteria have been fulfilled and the conclusions are likely or very likely to alter.

### Data synthesis strategy

Data were analysed using thematic synthesis methodology which is appropriate for synthesising data related to participant experiences (Thomas & Harden, 2008). There is no minimum number of studies required for qualitative evidence synthesis (Lewin et al., 2015), rather the richness of the data was considered, and whether the findings are supported across multiple studies. The synthesis strategy followed three stages (Thomas & Harden, 2008).

First, all text within the study ‘Results’ or ‘Findings’ sections was extracted for data synthesis to capture both author interpretations and participant quotations (mixed method studies used qualitative data only). Participant quotations identified in ‘Conclusion’ or ‘Discussion’ sections but not apparent in the ‘Results’ or ‘Findings’ section were also included in the analysis.

Second, data were imported to NVivo 14 (qualitative data analysis software) and then coded ‘line by line’ by the lead researcher. Codes were created to capture content as well as meaning, as per Thomas and Harden (2008) methodology. Codes were reused as appropriate, as well as new codes being created as the coding progressed, and the data were also re‐read after all initial coding had occurred, so that earlier codes could be applied as necessary.

Finally, the lead researcher grouped the codes into clusters, or descriptive themes, based on commonality; for example, a descriptive theme was created based on codes which described the relational aspects of working with HD. Greater weight was given to codes which were used across multiple papers. There were several codes which were created and used only once and could not be easily clustered together with other codes; these were therefore put into an ‘Other’ category.

The difference between descriptive themes and analytical themes can be summarised as descriptive themes captured interesting points of observation in the data, whereas analytical themes sought to answer the research question. Analytical themes also captured contrasting views in the data set and therefore synthesised the data rather than merely described it, aiming to capture the experiences of professionals and non‐professionals working with people with HD. Eight descriptive themes were created. For example, one descriptive theme was titled ‘Complexity and co‐morbidity in people with HD’. Moving from a descriptive theme to analytical required referring back to the research question, to consider what the experience of working with HD is like. Therefore, this descriptive theme became an analytical theme in the form of ‘Possessions are just the tip of the iceberg’. Subthemes were created by clustering together codes which were felt to capture the nuances of the findings; therefore, in what *way* are possessions just the tip of the iceberg?

### Reflexivity statement

The following definition of reflexivity was used in this systematic review: ‘Reflexivity is a form of critical thinking which aims to articulate the contexts that shape the processes of doing research and subsequently the knowledge produced’ (Lazard & McAvoy, 2020). A reflective diary and regular supervision discussions serve as formal methods of reflexivity to consider influences impacting the analysis of the extracted data. Thomas and Harden (2008) state that ‘…the idea or step of “going beyond” the content of the original studies…is the most difficult to describe…since it is dependent on the judgement and insights of the reviewers’. Therefore, reliability checks in the context of this qualitative synthesis were carried out as follows. Excerpts of the coding process were shared with J.G. (co‐author) at an early stage to ensure that codes could be understood as stand‐alone, meaning‐based items independently from the data, rather than simply data summaries. The iterations of descriptive and analytical themes were discussed with J.G. (co‐author), and the naming of themes and subthemes was discussed with J.G. and L.W. (co‐authors). The lead researcher was ‘closest’ to the data and therefore decided whether themes accurately captured what the data set as a whole was describing.

## RESULTS

### Study characteristics

Data from 12 studies (see Table 4) were included in the final analysis, consisting of nine published studies and three unpublished doctoral theses. All the studies were conducted in Western contexts: *n* = 7 studies were conducted in the United States, *n* = 4 in the United Kingdom, and *n* = 1 in Canada. Data were gathered from 150 participants (*n* = 137 professionals, *n* = 13 non‐professionals i.e. volunteers). Full details can be found in Table 4.

**TABLE 4:** Thematic synthesis summary table of included papers.

Author	Year	Country	Identification of HD	Participant role and key characteristics	Recruitment strategy	Data collection method	Study design and data analysis	Key findings
Ayers, Bratiotis, Saxena, & Wetherell	2012	The US	Hoarding Rating Scale (Tolin et al., 2010)	*n* = 1 licensed clinical psychologist with specialty training in geropsychology and hoarding, White female	Clinical psychologist whom had delivered the treatment (manualised CBT protocol for hoarding)	(1) clinician observation; (2) CBT consultant observation; (3) clinical treatment notes; (4) participant feedback; (5) participant in‐session notes and completed homework assignments	Qualitative analysis ‐ collective case study methodology	Themes of homework and treatment session compliance, skill deficits (prospective memory, planning, problem solving, and cognitive flexibility)
Holden, Kellett, Davies, & Scott	2019	The UK	Frost & Hartl's (1996) definition of hoarding, and homes would score ≥4 on Clutter Image Rating Scale (Frost et al., 2008)	Q‐sort completed by *n* = 36 professionals (*n* = 14 mental health, *n* = 19 housing, *n* = 1 environmental health, *n* = 2 fire fighters). Age range 26–61 years (M = 42.5, SD = 9.3); female = 22/36 (61.11%). Years in current occupation = 1–31 years (M = 10.7, SD = 7.7)	Participants were self‐selecting, the ‘presumed interest’ criteria to select participants was experience of working with people that hoard	Semi‐structured interviews with *n* = 5 professionals (consultant clinical psychologist, social worker, care manager in older adult mental health, housing officer, environmental health officer), analysed using thematic analysis which generated statements for Q‐sort task (*n* = 36) which was completed following a standard set of instructions	Q‐methodology	Three clusters of professionals identified: Cluster A = therapeutic and client focused, Cluster B = shocked and frustrated, Cluster C = pragmatic and task‐focused
Koenig, Leiste, Spano, & Chapin	2013	The US	Older adults with hoarding behaviours ‐ identified by a team member as hoarding inanimate objects and/or animals	*n* = 15 hoarding multidisciplinary team members, 12 women, 3 men. Age range 26–66 years (average 47 years), *n* = 6 AAA case manager, *n* = 1 APS director, *n* = 1 law enforcement officer, *n* = 1 AAA minor home repair manager, *n* = 1 AAA director, *n* = 1 animal control officer, *n* = 1 public health nurse, *n* = 1 AAA MSW student intern, *n* = 1 APS social worker, *n* = 1 mental health provider	Consultant panel used to identify members of multidisciplinary hoarding teams. Study participants were recruited from four teams	Semi‐structured telephone interviews lasting 40–90 min, *n* = 3 completed a second telephone interview lasting 15–30 min to confirm findings	Qualitative ‐ constant comparative method, grounded theory	Participants shared perspectives on (a) hoarding as a mental illness, (b) agency constraints for providing mental health services, and (c) the older adult's right to refuse these mental health services. They also outlined what makes for successful team work: (1) how well team members work together (2) agency policies (3) external support and (4) team members' capacities to develop trust with older adults
Koenig, Spano, Leiste, Holmes, & Macmillan	2014	The US	Older adults with hoarding behaviours ‐ identified by a team member as hoarding inanimate objects and/or animals in which the political approach (i.e., bargaining and/or coercion) had been used in 16 cases	*n* = 10 participants (7 = female, 3 = male). *n* = 3 AAA case manager, *n* = 1 APS director, *n* = 1 law enforcement officer, *n* = 1 animal control officer, *n* = 1 public health nurse, *n* = 1 AAA MSW student intern, *n* = 1 APS social worker, *n* = 1 mental health provider. Age range 26–66 years (average 44 years)	Consultant panel used to identify members of multidisciplinary hoarding teams. Study participants were recruited from four teams	Semi‐structured telephone interviews lasting 40–90 min, *n* = 3 completed a second telephone interview lasting 15–30 min to confirm findings	Qualitative ‐ constant comparative method, content analysis	Outcomes of these strategies resulted in a majority of older adults being removed from their home. Implications include the need for teams to increase understanding of their roles in service delivery; and additions to the political approach for addressing hoarding behaviours
Murdock (unpublished)	2006	The US	Identified as having significant clutter, inability to use living space, and impaired functioning due to accumulating objects of little usefulness	*n* = 5 adult protective services workers, must currently work in APS at least 50% of their time and be full‐time employees. Worked with between 1 and 16 hoarding cases over the last 2 years	Hoarding questionnaire posted to all adult protective services workers in Virginia. *n* = 5 randomly selected from 31 who volunteered to take part	Open‐ended telephone interview that lasted about 1 h	Mixed methods ‐ coded and allocated to pre‐determined categories	Workers described how hoarding was functional: emotional attachment to possessions, reducing stress, and providing meaning and identity. Hoarding often equated to addiction
Noyes, van Houten, & Wilkins	2024	The US	Unknown	*n* = 6 volunteers for Friendly Visitor intervention ‐ most were masters‐level occupational therapy students engaging in fieldwork experience, supervised by first author, unclear who remaining volunteers were. *n* = 4 female, *n* = 2 male. Age range = 20–29 years = 2, 30–39 years = 2, 50–59 years = 2	Flyers and emails from lists of volunteers and clients who had taken part in the Friendly Visitor programme within the first 2 years	Semi‐structured interviews	Qualitative ‐ codes and themes	Three themes: Relationship (sub‐themes: expectations and biases, the process of forming the relationship, and the outcome); Demands of decluttering (sub‐themes: physical demands, emotional demands, and cognitive demands); Strategies (sub‐themes: Consistency, Frequency, Duration, and Pacing; Having a Plan and a System; Making Decisions and Attending to the Task)
Porter & Hanson	2022	The UK	Clutter Image Rating Scale ‐ but not clear if specifically used for cases discussed	*n* = 11 staff from a Housing Department in a city council (housing officers, Specialist support officers, tenancy management and public protection), years of experience = 2–20 years, caseload of approximately 10–12 people with hoarding behaviours. Range of background such as mental health support, working with older people and environmental health	Email created by researchers was promoted by senior manager in Housing Department, staff to email to express interest and then sent further information	Approx. 60 min interviews via Microsoft Teams using interview guide by two female post‐doctoral researchers	Mixed methods ‐ Reflexive Thematic Analysis	Five themes: Working with others, Balancing an enforcement approach, Feeling conflicted, Complex needs of people who hoard and Staff needs
Ryninks, Wallace, & Gregory	2019	The UK	Clutter Image Rating Scale (Frost et al., 2008)	*n* = 7 volunteers, mean age 52.6 years, (SD 21.4), 2 = male, 5 = female, 4 = retired, 2 = full time employment, 1 = homemaker, 2 = A level or equivalent, 3 = Bachelor's degree, 2 = Master's degree	Through a UK‐based charity that provides advice and assistance to enable older and disabled people to continue living independently	All interviews were audio‐recorded with participants' consent and lasted between 40 and 60 min. The interviews were semi‐structured and aimed to elicit information about volunteers' experiences of providing help	Qualitative – IPA	Four superordinate themes: relationship between client and volunteer; ‘live life again’; challenges; and supporting volunteers. Eleven subordinate themes: Space to talk, Non‐professional status, Client‐led, Making space, Company and quality of life, Domino effect, Shame and embarrassment, Discarding possessions, Uncertainty, Training needs, Peer support
Tinlin	2022	The UK	Unknown	*n* = 30 clinicians working in NHS older adult mental health teams with experience of working clinically with older adults with hoarding disorder. 40% mental health nurses, 17% = psychologists, 17% = clinical support assistants, 16% = occupational health and physiotherapy, 10% = psychiatrists	Opportunistic sampling methods	Online survey ‐ qualitative data regarding clinician's subjective experiences of working with HD, guided by open ended questions	Mixed methods ‐ thematic analysis	Six themes outlining experiences of working with hoarding: (1) contextual factors (time constraints, scale of clutter, previous negative experiences influencing current treatment, lack of skills); (2) assessment (risk, life story, beliefs and motives, MDT); (3) formulation (co‐creation, motives and beliefs, helping staff understanding); (4) treatment (values based, support systems, discarding, meaningful activity, monitoring progress); (5) therapeutic relationship (trust, making the time, reinforcement); (6) multiagency working (skill profile, differing perspectives, coordination)
Vailu'u (unpublished)	2018	The US	Unknown	*n* = 11 social workers (*n* = 9 female, *n* = 2 male), and who maintain a caseload of older adult clients that include those receiving direct social service assistance for hoarding. Age range = 27–65+ years	Purposive sampling strategy ‐ information session scheduled with potential social work participants	×2 1 h focus groups ‐ original 6‐item semi‐structured qualitative interview schedule during the focus group process	Qualitative ‐ action research methodology, grounded theory. Constant comparative analysis technique	Themes identified: (a) barriers to mental health services, (b) funding and client financial constraints, (c) changes to current practice interventions, (d) practice challenges related to right to self‐determination, and (e) community education and support
Whitfield, Daniels, Flesaker, & Simmons	2012	Canada	Unknown	*n* = 10 members of community collaborative, representing a number of expert groups: social workers, home care nurses, geriatric neuropsychologists, geriatric nurses, fire and safety investigators, public health practitioners, and environmental health and safety officers	Unknown	Focus group ‐ face to face, approximately 1.5 h	Qualitative ‐ grounded theory	Themes related to community collaborative ‐ benefitted group members individually and collectively: Accessing expertise from the other group members; Maximising their own expertise; Enhancing their knowledge of hoarding behaviour
Yee (unpublished)	2020	The US	DSM‐5 criteria or as a symptom of OCPD	*n* = 8 somatically oriented psychotherapists or bodyworkers who have worked with clients on their hoarding behaviour	Criterion sampling, snowball sampling, systematic random sample	Online semi‐structured video interviews	Qualitative ‐ thematic analysis	2 major themes: (a) The clinician's therapeutic presence is the foundation of successful treatment, and (b) cultivating the client's mindfulness through interoceptive awareness led to unconscious materials.

### Quality appraisal

CASP quality appraisal ratings (CASP, 2024) are displayed in Table 5. Ten papers met all or most of the criteria, with two‐thirds of the papers giving inadequate consideration of the relationship between researcher and participants. Two papers met some of the criteria (Whitfield et al., 2012; Yee, 2020). One of these was an unpublished thesis (Yee, 2020) in which there were issues related to the rigour of the research (focus on professional experiences of working with HD which were lacking an evidence base), the statement of findings, and the value of the research given its lack of reference to current practice and policy.

**TABLE 5:** CASP quality appraisal ratings.

Author	1	2	3	4	5	6	7	8	9	10	NICE rating
Ayers et al. (2012)	Yes	Yes	Yes	Yes	Yes	No	No	Yes	Yes	Yes	++
Holden et al. (2019)	Yes	Yes	Yes	Can't tell	Yes	No	No	Yes	Yes	Yes	++
Koenig et al. (2013)	Yes	Yes	Yes	Yes	Yes	No	Yes	Yes	Yes	Yes	++
Koenig et al. (2014)	Yes	Yes	Yes	Yes	Yes	No	Yes	Yes	Yes	Yes	++
Murdock (2006)	Yes	Yes	Yes	Yes	Yes	No	Yes	Yes	Yes	Yes	++
Noyes et al. (2024)	Yes	Yes	Yes	Yes	Yes	Yes	Yes	Yes	Yes	Yes	++
Porter and Hanson (2022)	Yes	Yes	Yes	Yes	Yes	Yes	Yes	Yes	Yes	Yes	++
Ryninks et al. (2019)	Yes	Yes	Yes	Yes	Yes	No	Yes	Yes	Yes	Yes	++
Tinlin (2022)	Yes	Yes	Yes	Yes	Yes	Yes	Yes	Yes	Yes	Yes	++
Vailu'u (2018)	Yes	Yes	Yes	Yes	Yes	No	Yes	Yes	Yes	Yes	++
Whitfield et al. (2012)	Yes	Yes	Yes	Yes	Yes	No	Yes	Can't tell	Yes	Yes	+
Yee (2020)	Yes	Yes	Yes	Yes	Yes	Yes	Yes	No	No	No	+

### Thematic synthesis

Four analytical themes were identified: (1) Relationships are Complex; (2) Possessions are Just the Tip of the Iceberg; (3) Juggling Dilemmas on a Tightrope and (4) Working with Hoarding is Like a Puzzle to Solve. A summary of analytical themes and subthemes is outlined in Table 6. Themes are not inclusive of all codes but represent significant themes generated from the data set with the aim of answering the research question.

**TABLE 6:** Summary of Thematic Synthesis Analytical Themes and Subthemes.

Themes	Subthemes
Relationships are Complex	Removing Threat to Build Trust Trust is Necessary (But Not Sufficient) Others Can be a Help or a Hindrance
Possessions are Just the Tip of the Iceberg	It Takes a Physical Toll for Everyone The Emotional Overwhelm Cognitive Functioning Impacts Upon Work Trauma Takes Up Extra Space
Juggling Dilemmas on a Tightrope	It's Everyone's Job and No‐One's Job Choice is a Double‐Edged Sword
Working with Hoarding is Like a Puzzle to Solve	This Puzzle Feels Unsolvable The Intrigue of the Puzzle Needing to Think Outside the Box

#### Relationships are Complex

This theme describes the relational difficulties experienced when working with people with HD, including the role of trust in underpinning the work and the consideration needed to be given to external relationships. People with HD report having less social support and higher levels of loneliness compared to individuals with OCD (Edwards et al., 2023), and it appears that people working with HD are attuned to this.

##### Removing Threat to Build Trust

Professionals understood that they were likely to be perceived as a threatening presence to people with HD and that it is necessary to change this perception, stating ‘We have to prove we are not a threat and won't get rid of their possessions without permission before we can start anything’ (Tinlin, 2022). They also understood it was important to acknowledge the lack of trust people have in professionals: ‘It does him a great disservice if you go and try to deal with his hoarding, without dealing with his lack of trust’ (Yee, 2020). Non‐professionals were able to appear less threatening through self‐disclosure; a volunteer participant from Ryninks et al. (2019) described ‘Having that relationship and similar life experiences, we've been able to engage with each other… I do think that me being myself helps a lot’. The ability of volunteers to build trust may also be due to volunteers having chosen to support individuals with HD as opposed to professional duty, as well as professionals having the ability to apply statutory powers in contrast to volunteers.

##### Trust is Necessary (But Not Sufficient)

Professionals recognised that even when trust is in place, this is not always enough for a successful intervention; this was captured by Koenig et al. (2014) who stated ‘the team's establishment of a trusting relationship did not necessarily lead to a reduction in hoarding behaviors’. Development of trust was also affected by past interactions with services as described by Tinlin (2022): ‘…clinicians described how service users' previous negative experiences of accessing services can impact their interest in engaging with services and their ability to trust professionals’. The findings are also consistent with the reports of help‐seeking in people with HD, with this theme corroborating the importance of needing to build trust with the individuals they are supporting (Brown & Pain, 2014).

##### Others Can be a Help or a Hindrance

It was apparent across studies that professionals were aware that relationships with others could be a help or a hindrance for the person with HD. They recognised that people were often socially isolated, but that not all relationships proved to be supportive: ‘There was an awareness that involving friends and families alongside the client could be either positive or problematic and required careful consideration’ (Porter & Hanson, 2022). Murdock (2006) also stated in relation to APS (Adult Protective Services) workers that ‘…one common theme is that the hoarders are socially isolated, that they have conflictual relationships with children or spouses, if they have any contact at all, and some of the hoarders have no outside contact’. Therefore, promotion of external relationships which are supportive for the individual may help to bolster professional and non‐professional intervention.

#### Possessions are Just the Tip of the Iceberg

This theme reflects the complexity in working with people with HD, which occurs across a number of domains.

##### It Takes a Physical Toll for Everyone

This subtheme reflects the physical challenges of the work. Professionals and non‐professionals reported that people with HD often presented with a number of comorbid physical health problems. Volunteers in particular described how these physical health problems subsequently made the work more challenging; for example, ‘Her main barrier was her health and her ability to move, so trying to encourage her to make space during the week when I wasn't there is not something that she could do. So I think her health and mobility was, yeah, a real barrier for her’ (Ryninks et al., 2019). However, volunteers also experienced their own physical health problems which affected their ability to engage in the work, especially given that the voluntary work was primarily taking place in the person with HD's home: ‘I have asthma, and dust is an irritant for me, so I had a lot of trouble breathing. So that was sort of a barrier’ (Noyes et al., 2024), and ‘My hearing isn't absolutely perfect so it was slightly difficult to engage with him’ (Ryninks et al., 2019). The challenge of physical health problems may at least partially explain why people with HD report being only moderately satisfied with volunteer support in the group CBT for HD programme (Crone et al., 2020).

##### The Emotional Overwhelm

This subtheme describes that a significant aspect of the work is managing the emotional impact upon the individual with HD, mainly in relation to discarding: Murdock (2006) stated that ‘Workers, particularly Anne, explained that clients are anxious and emotional when their possessions are discarded’. A volunteer also reported ‘even the smallest bit that left her house was still an emotional process for her, even a small amount was huge work’ (Noyes et al., 2024). Sometimes professionals recognised that others may be better placed to offer emotional support, stating ‘the professional organizer…can now maximize her cleaning and organizational skills while directing clients' emotional issues to a trained professional’ (Whitfield et al., 2012). Importantly, there was also an emotional impact upon the worker themselves. Volunteers stated ‘Sally…generalized what many of the volunteers reported’: ‘It was a lot more emotional than I had anticipated because, for me, I could feel the agony for her of making these decisions’ (Noyes et al., 2024). Again, this appeared pertinent for volunteers due to them working among the hoard and being faced with the task of sorting and discarding. Professionals may be emotionally overwhelmed in relation to other aspects of the work, such as that caused by the ethical dilemmas presented in their role and feelings related to being stuck in their work.

##### Cognitive Functioning Impacts Upon Work

Professionals and non‐professionals reported a range of cognitive functioning issues observed whilst supporting people with HD, which contributed to the difficulties experienced. This was particularly noticeable in the context of CBT sessions, with the therapist reporting problems with ‘…the acquisition of new information, retention of newly acquired information, attention, response inhibition, decision making, categorization, organization of information, and planning’ as well as cognitive inflexibility ‘…even when presented with evidence showing that their methods were not working’ (Ayers et al., 2012). Similar deficits were also apparent in volunteer‐led interventions: ‘Participants also noted difficulty with planning, thinking ahead, organizing, and keeping on track while decluttering’ (Noyes et al., 2024). Cognitive difficulties in HD can therefore be anticipated from the outset and will need to be viewed in terms of the impact this may have on an individual's ability to engage with the intervention as well as considering adaptations which may be needed.

##### Trauma Takes Up Extra Space

The role of trauma was acknowledged in a number of ways across the studies. There were numerous references to the trauma histories experienced by people with HD, which was captured by Yee (2020): ‘The variety of experiences includes physical, emotional, and sexual abuse; extreme poverty; home relocation; complex grief; and natural disasters. Most participants reported more than one trauma for their clients’. There was some agreement among professionals that trauma may have precipitated HD and therefore trauma would need to be addressed first: ‘Since there was strong correlation of the location of hoard and the location where the trauma took place, which was in her room, until the trauma is resolved, the hoard may remain’ (Yee, 2020). A participant from Porter and Hanson (2022) also stated: ‘I think of our experience of hoarding cases as there some level of trauma that's been a trigger for why they hoard…And you know, dealing with and processing that trauma. That might need to come first before we even touch on the hoarding, because there's a reason why all their stuff is there’ (p. 2296). This raises a concern that particularly volunteers working with HD, without the support of a multidisciplinary team, may feel out of their depth, reflected in volunteers expressing a desire for more supervision (Noyes et al., 2024; Ryninks et al., 2019).

#### Juggling Dilemmas on a Tightrope

This theme describes the balancing act of working with HD and the conflicts which people face in their line of work.

##### It's Everyone's Job and No‐One's Job

Professionals often described challenges in relation to working with other services and understanding who is taking ownership of HD. Most appeared aware of the remit of their role, stating for example ‘I mean we are not social workers, we're not mental health workers, we are public health inspectors’ (Whitfield et al., 2012) and ‘Each of us know what we can and we can't do’ (Koenig et al., 2013). However, there were issues such as ‘…difficulty making appropriate referrals (e.g., mental health), a lack of understanding across services about roles and responsibilities and a need for a more collective approach’ (Porter & Hanson, 2022). Volunteers did not report these same challenges, perhaps because their remit was more clearly defined with less responsibility compared to professionals for ensuring services are in place.

##### Choice is a Double‐Edged Sword

Professionals recognised that it was important for clients to retain autonomy but that there were associated difficulties with offering help in this context. For example, a participant from Vailu'u (2018) described ‘I think one of the biggest challenges is…you shouldn't live like this, but then that's their choice…if they have capacity, how can we help someone who doesn't think they have an issue…so that's where it is hard’. Koenig et al. (2013) also said ‘Four [participants] stated that if the older adult as hoarder is “still competent and shows that she is of sound mind, she's entitled to make bad decisions. That is totally her choice to have that home environment’. This demonstrates the lack of insight which can be apparent in working with people with HD (Frost et al., 2010) and the subsequent difficulties in offering meaningful support to the individual.

#### Working with Hoarding is like a Puzzle to Solve

This theme describes the range of approaches taken by professionals and non‐professionals in approaching their work with HD. Individuals with HD report wanting professionals to have a better understanding of the problem (French et al., 2022), and this theme is reflective of the perceived lack of knowledge professionals and non‐professionals experience in working with this client group.

##### This Puzzle Feels Unsolvable

This subtheme reflects a group of professionals who feel stuck in their work with HD, describing how it is ‘… “impossible to know where to start”’ (Tinlin, 2022) and how daunting the prospect of the work feels: ‘I have at times felt frightened of the extent to what I'm working with’ (Porter & Hanson, 2022). There was also a sense of hopelessness described; for example, a participant from Koenig et al. (2013) reported ‘That's why I don't feel successful with hoarding situations. I've never felt like I've changed their hoarding behaviour; all that's happened is that their condition has declined, or…some kind of incident has forced them out of the situation’. It is important to note that staff perceptions of some mental health diagnoses can negatively affect subsequent behaviour; for example, mental health workers holding negative perceptions of borderline personality disorder can lead to reduced empathy and more rejecting behaviours towards individuals with this diagnosis (McKenzie et al., 2022). Therefore, the perception of working with HD could similarly take the form of negative stereotypes which may subsequently impact upon the delivery of treatment.

##### The Intrigue of the Puzzle

This subtheme represents a group of professionals and non‐professionals who find working with people with HD to be appealing. Holden et al. (2019) described professionals as ‘fascinated by the processes that people who hoard use to justify keeping things’. Similarly, Murdock (2006) reported ‘She is very intrigued with the topic of hoarding, using words like “interesting” and “fascinating”’. This was also supported by a volunteer perspective: ‘I actually am interested in, sort of, the psychology side of it…’ (Ryninks et al., 2019). For some, this may act as a counterbalance to the challenges of the work, and may be what drew people, particularly volunteers, to the work in the first place.

##### Needing to Think Outside of the Box

This subtheme refers to the realisation that professionals and non‐professionals demonstrated in needing to think differently when working with HD, in particular, changing the pace of the work. The slowness of the work compared to working with other clinical groups was commonly referred to by professionals and non‐professionals. This pace appeared necessary to have any success in working with HD, but did not guarantee it. Professionals described that ‘…change can be a slow process’ (Holden et al., 2019) and ‘the slow and careful approach required with individuals with HD’ (Tinlin, 2022). A volunteer also stated, ‘It seemed simple, and maybe to outsiders it didn't look like we did much…and maybe gave her confidence that she is able to take care of herself and her environment even if it was small, small steps’ (Noyes et al., 2024). Professionals and non‐professionals also discovered that the work was slow which was not apparent from the outset. A participant from Yee (2020) stated ‘I didn't know! What the hell am I doing? This is ridiculous. Now I think, “Oh my goodness, this is just a very slow long game that I'm working with…I'm going to be really slow and strong with her”’. One volunteer stated, ‘I knew that it would be a process, I just didn't realize it would be so real…something as simple as a receipt would take this much time, thought, and processing…so much meaning was held in that receipt’ (Noyes et al., 2024). Professionals may benefit from drawing parallels with voluntary work, such as working in the client's home and offering a flexible number of sessions, which people with HD have reported as being helpful (Crone et al., 2020).

## DISCUSSION

The aim of the present review was to identify and synthesise professionals' and non‐professionals' experiences of working with people with HD. Four themes were identified: (1) Relationships are Complex; (2) Possessions are Just the Tip of the Iceberg; (3) Juggling Dilemmas on a Tightrope and (4) Working with Hoarding is Like a Puzzle to Solve. To the authors' knowledge, this is the first review to synthesise these experiences and to include both professional and non‐professional perspectives. Overall, the findings describe the experience of working with HD as being challenging for both professionals and non‐professionals, with professionals often feeling stuck in their work and faced with difficult decisions to make, compared to non‐professionals who are exposed to the practical challenges which arise from being in the client's home to support with sorting and discarding and being witness to the emotional impact of this.

The existing literature identifies low rates of clinically significant change for both group and individual CBT (Tolin et al., 2015), as well as problems with high drop‐out rates (Mataix‐Cols et al., 2002; Tolin et al., 2007), ambivalence towards treatment (Steketee et al., 2010), and poor motivation (Ayers et al., 2015). The thematic synthesis findings are consistent with these outcomes, in which the complexity of working with HD was widely recognised across both professionals and non‐professionals. There was also consensus across professionals and non‐professionals about the importance of needing to build trust and develop a relationship with individuals with HD, which is encouraging given that people with HD have expressed their difficulty in trusting others (McGrath et al., 2024). There was a lack of evidence that non‐professionals are afflicted by the same dilemmas as professionals, which can be understood in terms of the difference in role and responsibility. It was also apparent that some professionals and non‐professionals found the work to be intriguing. However, it is important to note that feeling stuck in the work and with the additional complexities related to cognitive functioning and history of trauma, there may be a risk of professionals applying negative stereotypes to people with HD, with stereotypes forming a component of stigma (Corrigan & Watson, 2002). For example, there is evidence that professionals perceive individuals with HD as more difficult to work with and are more likely to feel relieved when they do not attend appointments compared to non‐hoarding clients (Tolin et al., 2012).

The current study has practical implications both for those working with HD and for individuals themselves with these difficulties. The difficulties in multi‐agency working when supporting people with HD may be eased by deciding who is taking ownership of the case at an early stage in joint working and agreeing on the frequency of multidisciplinary meetings; stakeholders across a range of professional disciplines are supportive of a psychology‐led multi‐agency model (Haighton et al., 2023). Given the complexity involved in working with this client group, workers will need space to explore both the clinical and professional aspects of the work, but also countertransference, given the feelings of frustration which are reported in working with HD (Tolin et al., 2012).

Despite some people reporting feeling motivated and highly interested in the work, to the extent that they volunteer their free time, many appeared to feel out of their depth, overly responsible, and torn as to what to do for the best, which would be expected to negatively impact their own wellbeing (Meyer & Hünefeld, 2018). This would be consistent with the Job Demands–Resources Model of Burnout (Demerouti et al., 2001), with the interaction of high job demands and reduced external resources leading to burn out in the form of exhaustion and disengagement. This could be incredibly detrimental both for the wellbeing of helpers and for the people with HD who are so attuned to the importance of the therapeutic relationship.

The current review highlights the importance of providing effective clinical supervision and training in working with HD, for both professionals and non‐professionals. Effective supervision is vital for mental health workers across nursing, medics and allied health professionals to avoid burnout (Sellers et al., 2024) and similarly volunteers have also expressed a desire for this in the current study. It could be helpful for those working with HD to share anonymised examples of good practice and successful outcomes within their teams to help build hope that change can occur, whilst holding in mind that expectations of progress may differ from reality, as described by Noyes et al. (2024). This could help to promote more positive attitudes, dispel negative stereotypes and decrease the risk of stigma. Overlap also exists between professionals' frustration with gaps in service provision highlighted in the current study, which is also reported by people with HD (McGrath et al., 2024).

For those professionals who struggle to make progress when working with HD, it is important to communicate that there is a pre‐existing evidence base to refer to and that further training could increase confidence and skills. For example, it was notable that 13% of mental health professionals who participated in Tinlin's (2022) study reported being aware of the CBT model of HD but not making use of it in a clinical setting, and 95% of staff reported that they required training to work with HD. It is hoped that the consolidation of this evidence is validating for people working with HD and gives them an insight as to what to expect.

It is important for future research to understand whether perceptions of working with HD subsequently affect treatment outcomes, as well as investigate the impact of training and supervision upon helpers' attitude and knowledge. For example, providing helpers with a better idea of what to expect when working with HD, dispelling unhelpful narratives which may exist in relation to HD and identifying realistic outcomes, may help people to be better equipped in working with this client group which may have a positive impact upon the working relationship. It would also be helpful for future research to explore in more detail whether differences exist across professional disciplines. Future qualitative research would also need to consider quality aspects; for example, a number of the included studies did not adequately consider the relationship between researcher and participants, with reflexivity being critical to qualitative research (Shaw, 2010).

A strength of this review is that it offered a range of perspectives across helpers including professionals and volunteers, and it incorporated various professionals including those from mental health services, housing, and social work. Furthermore, most of the papers were rated as high quality. It should be acknowledged that given the thematic synthesis has consolidated both professionals' and non‐professionals' experiences, these experiences will vary. Despite this, the findings reflect that professionals and non‐professionals broadly experience similar challenges in supporting people with HD. There may however be differences in professional discipline due to the nature of contact; for example, differences may exist between housing officers and environmental health officers, given they may not be involved by choice of the individual, in comparison to clinical psychologists. From a methodological perspective, the potential for risk of bias could have been improved by having a 100% inter‐rating check at the article identification stage of the review process.

## CONCLUSION

To conclude, professionals and non‐professionals report experiencing challenges in working with HD across a number of areas. Both groups understand the importance of building trust with individuals with HD and are aware of factors that can make the work more complex. The work can present various dilemmas for professionals in particular, recognising that choice is important but can have detrimental consequences for the individual with HD. There were differing perceptions of the work, with some feeling hopeless and overwhelmed, and others intrigued by what drives hoarding behaviour, with this interest being especially apparent in volunteers. Professionals and non‐professionals would benefit from receiving tailored supervision and training to further their knowledge and confidence in working with HD.

## AUTHOR CONTRIBUTIONS

**Hannah Parker:** Conceptualization; writing – original draft; writing – review and editing; methodology; data curation; formal analysis. **Louise Waddington:** Conceptualization; methodology; supervision; writing – review and editing. **Bethan Shergold:** Data curation; writing – review and editing. **James D. Gregory:** Conceptualization; methodology; supervision; writing – review and editing; formal analysis.

## CONFLICT OF INTEREST STATEMENT

None of the authors have any conflict of interest to disclose.

## Data Availability

The data that support the findings of this study are available from the corresponding author upon reasonable request.
